# Point of care technology or standard laboratory service in an emergency department: is there a difference in time to action? A randomised trial

**DOI:** 10.1186/1757-7241-19-49

**Published:** 2011-09-10

**Authors:** Christian B Mogensen, Anders Borch, Ivan Brandslund

**Affiliations:** 1 Akutafdelingen, Kolding Sygehus, Kolding, Danmark; 2 Klinisk Biokemisk afdeling, Vejle Sygehus, Vejle, Danmark

## Abstract

**Background:**

Emergency Departments (ED) have a high flow of patients and time is often crucial. New technologies for laboratory analysis have been developed, including Point of Care Technologies (POCT), which can reduce the transport time and time of analysis significantly compared with central laboratory services. However, the question is if the time to clinical action is also reduced if a decisive laboratory answer is available during the first contact between the patient and doctor. The present study addresses this question: Does a laboratory answer, provided by POCT to the doctor who first attends the patient on admission, change the time to clinical decision in commonly occurring diseases in an ED compared with the traditional service from a central laboratory?

**Methods:**

We performed a randomised clinical trial with parallel design and allocation ratio 1:1. The eligibility Criteria were: All patients referred from General Practitioner or another referring doctor suspected for a deep venous thrombosis (DVT), acute coronary syndrome (ACS), acute appendicitis (AA) or acute infection (ABI). The outcome measure was the time spend from the blood sample was taken to a clinical decision was made.

**Results:**

The study period took place in October--November 2009 and from February to April 2010. 239 patients were eligible for the study. There was no difference between the groups suspected for DVT, ACS and AA, but a significant reduction in time for the ABI group (p:0.009), where the median time to decision was reduced from 7 hours and 33 minutes to 4 hours and 38 minutes when POCT was used. Only in the confirmation of ABI the time to action was significantly shorter.

**Conclusions:**

Fast laboratory answers by POCT in an ED reduce the time to clinical decision significantly for bacterial infections. We suggest further studies which include a sufficient number of patients on deep venous thrombosis, acute appendicitis and acute coronary syndrome.

## Background

The Emergency Departments (ED) are characterized by a high flow of patients with a broad range of different conditions and timely delivery of services is crucial to avoid congestion. In order to achieve a reduction in length of stay every step from admission to discharge must be optimized, including a reduction in waiting time for laboratory results.

New technologies for laboratory analysis have been developed, including Point of Care Technologies (POCT) [1]. These technologies ought to be faster and easier to use than the standard central laboratory, and still have a comparable quality of the results [2]. Such technologies are increasingly available and can reduce the transport time and time of analysis significantly compared with central laboratory services [3-5].

In an ED a plan of treatment for the patient often depends on a laboratory answer. In some cases a laboratory test directly determines the next step of a plan. The result of D-dimer guides the decision, if a patient suspected for deep venous thrombosis should have an ultrasonography scan of the leg performed [6]. For a patient with chest pain and a normal ECG the result of Troponin and Creatin Kinase directs the clinical action [7]. In other cases the laboratory results might be supportive for a clinical decision, like the CRP result to decide if a patient suspected of bacterial infection should start antibiotic treatment [8] or a patient, suspected for acute appendicitis should be operated [9].

A time reduction from a blood test is requested to the answer is available might be important. However, the crucial question is, if the time to clinical action is also reduced if a decisive laboratory answer is available during the first contact between the patient and doctor. Several other factors might influence, like interpretation of the laboratory result to the clinical presentation, the doctors level of experience, time allowed to attend the patient and waiting time for other investigations [10]. The present study addresses this question: Does a laboratory answer, provided by POCT to the doctor who first attends the patient on admission, change the time to clinical decision in commonly occurring diseases in an ED compared with the traditional service from a central laboratory?

## Methods

### Design

We performed a randomised clinical trial with parallel design and allocation ratio 1:1. The eligibility Criteria were: All patients referred from General Practitioner or another referring doctor suspected for a deep venous thrombosis (DVT), acute coronary syndrome (ACS), acute appendicitis (AA) or acute infection (ABI). These groups were chosen since they are common in the ED, and the clinical decision to be taken depends more or less on a laboratory tests. Even though most surgeons agree that the diagnosis of appendicitis is not very dependent on the CRP value, the result of inflammatory variables has a discriminatory value [9]. Appendicitis was included in the study, as it was an experience in the Kolding ED that most decisions on this diagnosis were made after the results of CRP were available.

The exclusion criteria were suspicion of ACS with ECG changes which demanded immediate clinical action (like ST-elevation) or other acute pathological ECG findings or previous ACS; suspicion of AA and ABI where the condition requires immediate action (like signs of severe peritonitis, severe sepsis or meningitis). The outcome measure was the time spend from the blood sample was taken to a clinical decision was made. The clinical decision was defined as follows: for DVT the decision of referring for ultrasonography or rejection of the suspicion of DVT; for the suspicion of ACS: the diagnosis was confirmed and the patient transferred to coronary care unit, or the ACS suspicion rejected; for the suspicion of AA: the decision of an operation or the diagnosis of AA rejected; for the suspicion of ABI: the decision of prescribing an antibiotic or the rejection of a bacterial cause of the infection. The time of decision was reported in minutes and was the time in the electronic patient file, which first indicated that a decision was made, either by a notice from a physician or nurse, a prescription of medicine or operation, a transfer to a coronary care unit or a request for ultrasonography.

There were four blocks of randomisation numbers, one for each diagnosis. For each diagnosis 48-52% were even numbers and used for the POCT analysis.

### Location

The study took place at the ED at Kolding Sygehus, Denmark. The ED received around 9.000 patients annually for admission with surgical, medical, cardiological or ortopaedic conditions. All patients were referred from a GP or another doctor outside the hospital.

Four research assistants were responsible for inclusion, registration, POCT- analysis and registration of outcome. The study was only opened for inclusion when one of the research assistants was available. The decisive blood test was D-Dimer for DVT, Troponin I and Creatin kinase- (CK-MB) for ACS (Troponin T at the central laboratory), and CRP for AA and ABI.

The POCT- analysis was performed in the AQT-90 (Radiometer). The AQT-90 is developed for high quality laboratory test and utilises a time-resolved fluorescence method to detect complexes formed between capture antibodies, fluorescent tracer antibodies, and the antigen of interest. The results are available after 15-20 minutes depending on the parameter analyzed. The AQT-90 analysis for TnI has been shown to be marginally inferior to two laboratory assays in diagnosing AMI [10], and comparable to standard laboratory assays in analysis of D-dimer for DVT [11].

All patients with suspected ACS 3 consecutive normal analysis of troponin were required before the diagnosis was rejected. Analysis of TnI at AQT-90 was only performed the first time, while the second and third analysis was ordered 6 and 12 hours after the admission from the central laboratory.

The Central laboratory used Modular E1-170 (Roche Diagnostics) for analysis of the blood samples. The results are available after 1-2 hours.

When a patient was referred with one of the presumed diagnosis of interest, the study assistant was called and performed the randomisation to POCT-analysis (intervention group) or to the standard central laboratory (control group). When the laboratory assistant draw the blood sample, the study assistant performed the POCT- analysis if the patient was randomised to POCT-arm, otherwise the blood sample was transferred to the central laboratory.

The physician, who admitted the patient, received the POCT laboratory test answer slip from the research assistant during the admission procedure. The answers from the central laboratory appeared in the electronic patient file, and it was the duty of the admitting physician to trace the results in the file.

The admitting physician was normally an ED employed doctor, often newly graduated and with few months of experience. A range of specialist was available for consultation at all times.

### Statistics and ethics

The sample size calculation estimated 2 × 35 patients for each diagnose, assuming a time to decision of 240 minutes, with a SD of 60 minutes, a 1% risk of type I error and 5% risk of type II error and a minimum relevant difference of 60 minutes between the two groups.

All results were recorded on preprinted forms, transferred to electronic form by using Epidata 3.1. and analyzed in STATA version 7. Continuous data are presented as medians with inter-quartile ranges and compared with the non-parametric Mann-Whitney U -test. Categorical data are presented with a number and percentage of occurrences and compared with Fishers exact test.

After contact to the regional ethic committee, no approval was required for this study. Since it was a study of a working method, not related to any contact with the patient or included any additional test to the routine for the patient, no information to the patient or consent was required nor registration at a public trial registry. The study was registered at the Danish Data Protection Agency (J.nr. 2009-41-3923).

## Results

The study took place in October--November 2009, and from February to April 2010. 239 patients were eligible for the study. The mean age of the patients in the POCT group was 50.9 years versus 51.5 years in the central laboratory group (p: 0.83) and 58% were females in the POCT group versus 42% in the control group, a non-significant difference (p: 0.08). The randomisation time of the day was equally distributed in both groups (p: 0.26). Seven patients were excluded because a definite endpoint could not be identified.

**Figure 1:**
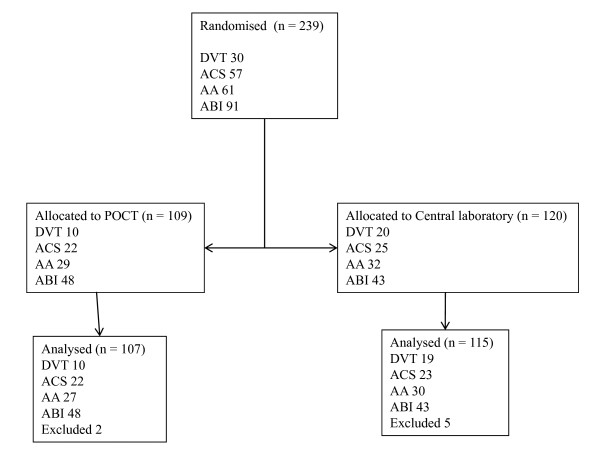
**Flow diagram for the trial**. Abbrivations: POCT: point of care technology; DVT: deep venous thrombosis; ACS: acute coronary syndrome; AA: acute appendicitis; ABI: acute bacterial infection.

The distribution between the different randomised groups is shown in Figure 1.

In table 1 the time to clinical action is calculated. There was no difference within the groups suspected for DVT, ACS and AA, but a significant reduction in time for

**Table 1:** Central laboratory vs POCT: time to action in an Emergency Department

Condition	group	number	median (minutes)	p25 (minutes)	p75 (minutes)	p-value*
Observation for deep venous thrombosis (DVT)	central lab.	19	282	183	425	0.91
	POCT	10	316	180	477	
	difference		34			
Observation for acute coronary syndrome (ACS)	central lab.	23	757	365	1285	0.75
	POCT	22	708	217	1226	
	difference		-49			
Observation for acute appendicitis (AA)	central lab.	30	207	137	388	0.98
	POCT	27	247	130	384	
	difference		40			
Observation for acute bacterial infection (ABI)	central lab.	43	453	257	1127	0.009
	POCT	48	278	123	598	
	difference		-175			

*Mann- Whitney test

the ABI group (p:0.009), where the median time to decision was reduced from 7 hours and 33 minutes to 4 hours and 38 minutes when POCT was used.

In table 2 the time to rejected or confirmed diagnosis is calculated, depending on the laboratory technology used. Only in the confirmation of ABI the time to action is significantly shorter when POCT is used.

**Table 2:** time to action in confirmed and rejected diagnosis

condition	diagnosis	group	number	median time (minutes)	p25 (minutes)	p75 (minutes)	p-value*
Observation for deep venous thrombosis (DVT)	confirmed	Central lab.	3	313	273	344	0.14
		POCT	2	354	344	366	
		difference		41			
							
	rejected	Central lab.	16	273	166	428	0.81
		POCT	8	242	121	504	
		difference		-31			
Observation for acute coronary syndrome (ACS)	confirmed	Central lab.	2	1566	365	2767	0.12
		POCT	2	188	158	217	
		difference		-1378			
	rejected	Central lab.	21	757	422	1264	0.86
		POCT	20	853	483	1248	
		difference		96			
Observation for acute Appendicitis (AA)	confirmed	Central lab.	4	185	119	261	0.87
		POCT	8	214	123	309	
		difference		29			
	rejected	Central lab.	26	234	137	415	0.85
		POCT	19	251	171	478	
		difference		17			
Observation for acute bacterial infection (ABI)	confirmed	Central lab	31	399	257	972	0.02
		POCT	34	237	115	489	
		difference		-162			
	rejected	Central lab	12	929	247	1441	0.23
		POCT	14	281	156	743	
		difference		-648			

* Mann-Whitney test

## Discussion

We found a significant reduction in time to action of approximately 3 hours for patients suspected for acute bacterial infection. It was for the confirmed diagnosis of ABI that the POCT reduced the time to decision. It was not possible to reach a conclusive number of patients for the AA, DVT and ACS groups. For AA there was a non-significant tendency for the POCT analysis to increase the time to decision.

An acute bacterial infection has often developed for days at the time of admission, and is accompanied with obvious focal sings of infection. It is possible for even a newly graduated doctor to make decisions about antibiotic treatment for the majority of cases suspected for a bacterial infection with a confirmatory CRP result.

In the diagnosis of appendicitis observation time is important and repeated abdominal examinations are often necessary. Diagnosing appendicitis requires long experience and clinical skills beyond the level of a newly graduated ED doctor and the diagnose is not dependent on the CRP result alone [9].

Several other recent POCT studies have been reported, especially on the suspicion of ACS. In a French randomised study similar to ours, it was found that POCT in an emergency department reduced the time to anti-ischaemic therapy significantly, but not the length of stay in the ED in the hands of emergency physicians [12]. Singer AJ et al (2008) reported similar results from US [13] while a pre- post POCT study from Boston (2003) showed a non-significant reduction in length of stay for cardiac markers if measured by POCT [14]. However, we did not find a study which compared a range of different high quality POCT results with the time to clinical action in an ED comparable to a Danish setting.

The study was weakened by the number of patients participating, which was too low to reach conclusions on some of the diagnosis of interest with a risk of type II error. The pre-study power calculation was based on clinical assumption of the time to diagnosis, which were too optimistic and the standard deviation showed to be far longer than the estimated 60 minutes.

We performed randomisation but had no clinical data reflecting if the groups were clinically comparable, which might not be the case in small groups and hence introduce an incidental skewness. Despite the randomisation procedure aimed at a distribution between the two groups of 48-52% almost 67% of the 29 DVT suspected patients' blood tests were examined in the central laboratory. This might have added to the skewness in the group. For the patients suspected for ACS 3 blood samples were taken with 6 hours interval to exclude ACS. Only the first blood sample was analysed on the POCT because it is not necessary to use POCT for a test which is taken as a routine or planned to be taken several hours after admission. Since the majority of ACS suspected patients needs two or three laboratory tests before a conclusion can be made, POCT will only be of limited value in these cases.

The study was interrupted around December- January for both groups. We do not believe that this had any impact on the results of the study. The study was not blinded, which might influence on the involved physicians decisions and recording of their decisions. The POCT answers were given directly to the physician caring for the patient, while it was the physician who had to trace the central laboratory result in the patient- file. This might give an additional delay in the time to action for the control group. However, the advantage of the POCT is not only short time of analysis but also the immediate access to the results.

The endpoint in this study was sometimes difficult to define, e.g. the time when an action was taken or a suspected diagnosis was rejected. However, it reflects the real life situation, and the problem is equally distributed in both the POCT and the control group, since it is not related to the laboratory technique.

In this study a study assistant without other assignments handled the POCT analysis. In real life a staff member might have other assignments in addition to the POCT analysis, which will prolong the time to the POCT answer. Furthermore the central laboratory was placed around 300 meters away. If transport time to the central laboratory is reduced this will reduce the difference in turnaround time between POCT and central laboratory.

## Conclusions

From our study we conclude, that fast laboratory answers by POCT in an ED reduces the time to clinical decision significantly for confirmed bacterial infections and suggest further studies which include a sufficient number of patients on deep venous thrombosis, acute appendicitis and acute coronary syndrome.

## Competing interests

The study was financially supported from Kolding Sygehus research foundation. The test supplies for the POCT analysis were provided from Radiometer, Denmark. The company did not have any influence on the study design or interpretation of the results.

## Authors' contributions

CBM concepted the idea for the study, assisted by IB in design. AB participated with CBM in the acquisition of data, which was analyzed by CBM and interpreted by all three authors. CBM drafted the manuscript which was revised by IB and AB. All three authors have given final approval of the version to be published.
